# Immunovirotherapy Based on Recombinant Vesicular Stomatitis Virus: Where Are We?

**DOI:** 10.3389/fimmu.2022.898631

**Published:** 2022-06-28

**Authors:** Yuguo Zhang, Bolni Marius Nagalo

**Affiliations:** ^1^Department of Pathology, University of Arkansas for Medical Sciences, Little Rock, AR, United States; ^2^Winthrop P. Rockefeller Cancer Institute, University of Arkansas for Medical Sciences, Little Rock, AR, United States

**Keywords:** vesicular stomatitis virus, oncolytic virus, genetically modified virus, cancer therapy, immunotherapy

## Abstract

Vesicular stomatitis virus (VSV), a negative-strand RNA virus of the *Vesiculovirus* genus, has demonstrated encouraging anti-neoplastic activity across multiple human cancer types. VSV is particularly attractive as an oncolytic agent because of its broad tropism, fast replication kinetics, and amenability to genetic manipulations. Furthermore, VSV-induced oncolysis can elicit a potent antitumor cytotoxic T-cell response to viral proteins and tumor-associated antigens, resulting in a long-lasting antitumor effect. Because of this multifaceted immunomodulatory property, VSV was investigated extensively as an immunovirotherapy alone or combined with other anticancer modalities, such as immune checkpoint blockade. Despite these recent opportunities to delineate synergistic and additive antitumor effects with existing anticancer therapies, FDA approval for the use of oncolytic VSV in humans has not yet been granted. This mini-review discusses factors that have prompted the use of VSV as an immunovirotherapy in human cancers and provides insights into future perspectives and research areas to improve VSV-based oncotherapy.

## Introduction

Vesicular stomatitis virus (VSV) is non-pathogenic, enveloped, negative-strand RNA *Rhabdovirus* with potent vaccine and oncolytic potential (1–6). VSV can infect nearly all cell types but cannot initiate a productive infection in healthy cells due to an antiviral response mediated by type-I interferons (IFNs).^11^ However, defects in IFN signaling often coincide with tumorigenesis (7, 8). Thus, VSV is capable of infecting and selectively destroying cancer cells with minimal damage to normal cells, making it an attractive therapeutic agent. Furthermore, VSV is particularly appealing as an oncolytic vector (OV) and vaccine agent due to low anti-VSV immunity in the general population (pre-existing immunity to OVs limits their intratumoral spread) and fast replication kinetics in cancer cells (9).

The nonsegmented VSV genome is typical of viruses in the *Vesiculovirus* genus. The approximately 11-kb genome encodes five structural proteins, including the nucleocapsid protein (N), phosphoprotein (P), matrix protein (M), glycoprotein (G), and the large polymerase protein (L) (10–12). VSV genome encapsidation is facilitated by specific interactions between the N and P proteins (12). The N protein is essential for suppressing the transcription-termination signal during viral replication (13). The P and L proteins function as co-factors of the RNA-dependent RNA polymerase (RdRp); they exert indispensable and versatile functions, including regulating the initiation, elongation, and encapsidation of viral RNAs (14, 15). Specifically, the RdRp binds the encapsidated viral genome at the leader region, then sequentially transcribes each gene (16).

The M protein is involved in virus assembly and budding (17). It has also been shown to inhibit innate antiviral responses and alter host transcriptional machinery, ultimately coercing tumor cells to undergo apoptosis (18). Therefore, viruses with mutant M proteins were developed to restrict viral replication to tumor cells with an altered type-I IFN signaling axis (19).

The G protein forms spike-like structures on the viral particle surface and plays an essential role in the initial stages of infection (20). In the Indiana strain, the G protein was shown to mediate viral attachment *via* interaction with the low-density lipoprotein receptor (LDL-R) and its family members (21). The VSV-G protein is capable of binding LDL-R *via* the cysteine-rich LDL-R domains CR2 and CR3, resulting in clathrin-dependent endocytosis and intracellular uptake of the VSV genome (22, 23). However, several other reports showed that isogenic pairs of wild type LDL-R and LDL-R–knockout (-/-) (24, 25) cell lines can be infected efficiently by VSV and other closely related family members, highlighting the potential role of other surface proteins or cell-intrinsic mechanisms in viral entry (22). The broad cellular tropism of VSV is attributable to its G protein; thus, it is often replaced with entry proteins from other viruses to improve the safety and selectivity of VSV-based oncolytic vectors (26).

Numerous studies have shed light on the fundamental mechanisms of VSV–host cell interactions, the dynamics of viral gene expression, and the pathogenesis of viral infection (26). These findings have greatly expanded our understanding of the biology and structure of VSV, informing the design of recombinant VSV (rVSV) vectors with improved safety and selectivity towards a broad range of cancer cells. Despite this progress, VSV-based immunovirotherapy has not lived up to its expectations, and FDA approval has not yet been granted. Thus, we eagerly await the published outcomes of various completed, recruiting, or active cancer treatment trials (clinicaltrials.gov) in the United States using rVSV as an immunovirotherapy platform. In the meantime, it is equally important to review the past and recent developments of VSV vectors in cancer therapy to derive insights into ways to refine and improve the antitumor efficacy of such vectors.

## Development of VSV as a Vaccine Platform

The use of reverse genetics enabled researchers to rescue infectious negative-strand RNA viruses from viral genomic cDNAs, leading to significant improvement in our ability to manipulate and study RNA viruses for vaccine development and cancer therapy applications (27, 28). Owing to their ability to prime robust humoral and cellular immunity, VSV vectors have also been used as vaccine agents to generate protective immunity against infections with highly lethal human viruses, including Ebola, HIV, Marburg, Lassa, Zika, and SARS-COV-2 viruses (29–40). Other vaccine candidates using attenuated VSV vectors were evaluated in preclinical models to prevent illnesses due to influenza (41), hepatitis B virus (42), different strains of coronavirus causing respiratory diseases (43, 44), *Yersinia pestis* (bubonic plague) (45), respiratory syncytial virus (RSV) (46), herpes simplex virus 2 (HSV2) (47), Dengue virus (48), Chikungunya virus (49), Nipah virus (50), and human papillomavirus (HPV) (51).

However, despite abundant evidence of therapeutic efficacy, only one VSV-based vaccine is FDA approved (52). This is mainly due to concerns related to the promiscuous nature of the VSV entry glycoprotein (VSV-G), allowing the virus to infect neurons and induce encephalitis in mice (7, 53, 54). Thereby, questions were raised regarding the potential neurotoxicity of VSV in humans following systemic delivery, limiting its widespread clinical deployment as a vaccine vector in humans. To address this critical concern, several groups have engineered VSV vectors with mutated G proteins or harboring G proteins from other non-neurotropic viruses to ablate interactions with LDL-R, which is highly expressed in neurons (49, 55–57). Many VSV-derived vectors that have progressed to preclinical and clinical testing as vaccine agents also displayed lytic potency and elicited a strong, durable cytotoxic T-cell response in permissive tumors (58, 59). While most oncolytic viruses such as VSV induce robust tumor-cell killing *in vitro*, recent clinical reports strongly suggest that, *in vivo*, OVs turn “cold” tumors into hot tumors (60), as discussed below.

## Rationale for Developing VSV as an Oncolytic Agent

Wild type VSV causes mild disease in cattle, horses, and swine, causing vesicles (blisters) around the mouth (61). The few reported cases of human VSV infections were limited to agricultural and laboratory workers, characterized by an incubation period between 8 to 48 hours, with mild flu-like symptoms (26, 62, 63). VSV is a highly cytopathic virus that infects nearly all cell types, but its infection and replication are enhanced in tumor cells with a defective IFN signaling pathway (64). This feature makes it an ideal oncolytic virus therapy agent. In addition, VSV has a fast kinetic cycle, does not integrate into the host genome (65), and is a potent inducer of apoptosis in the infected cancer cells—a critical feature of viral therapeutics (66, 67). The VSV genome is also relatively small and can accommodate the insertion of one or more foreign, functional genes (68). Importantly, VSV has demonstrated anticancer activity in a vast array of cancer cells, including osteosarcoma (69), cervical cancer (70), breast cancer (71), melanoma (72), hepatocellular carcinoma (73), pancreatic cancer (57), and glioblastoma (74).

Although VSV-based oncolytic vectors have shown efficacy in mouse models and led to multiple human studies (**Table 1**), barriers to FDA approval and clinical application remain. These barriers include variability in the efficiency by which VSV kills cancer cells, even among cancers from the same tissue of origin,^4^ and reports of VSV-induced encephalitis in laboratory animals and humans (87, 88). Furthermore, the heterogeneous therapeutic responses in solid cancers (e.g., pancreatic cancer) are attributed to factors such as a fibrotic and dense extracellular matrix, hypoxia, high interstitial tumor pressure, and low pH in the tumor microenvironment, limiting viral spread and immunogenic cell death in response to oncolytic therapy.^13^ The fact that VSV is cleared rapidly by the immune system (e.g., *via* neutralizing antibodies and complement molecules) has further dampened enthusiasm for this vector (7, 53, 54). These obstacles have severely limited the anticancer efficacy of VSV—particularly the inability to administer multiple doses to achieve tumor shrinkage and, most importantly, the inability to bypass the immune system and infect neoplastic cells.

**Table 1:** Reported VSV-based vaccine and cancer treatment clinical trials (http://clinicaltrial.gov/).

Vector	Purpose	Clinical Trial	Identification	Phase	Status
VSV-IFNβ-NIS	Cancer treatment	Systemic VSV-IFNβ-NIS and Pembrolizumab in Refractory NSCLC and NEC	NCT03647163	I/II	Recruiting**
VSV-IFNβ	Cancer treatment	Administration of VSV-IFNβ-NIS Monotherapy and in Combination With Avelumab in Pts With Refractory Solid Tumors	NCT02923466	I	Active not recruiting**
VSV-IFNbetaTYRP1	Cancer treatment	Modified Virus VSV-IFNbetaTYRP1 in Treating Patients With Stage III-IV Melanoma	NCT03865212	I	Recruiting**
VSV-GP	Cancer treatment	Phase 1b Study to Evaluate ATP128, VSV-GP128 and BI 754091 in Patients With Stage IV Colorectal Cancer	NCT04046445	I	Recruiting**
VSV-IFNβ-NIS	Cancer treatment	Intratumoral Administration of Recombinant VSV in Patients With Refractory Solid Tumors	NCT02923466	I	Recruiting**
VSV-IFNβ-NIS	Cancer treatment	VSV-IFNβ-NIS With or Without Ruxolitinib Phosphate in Treating Patients With Stage IV or Recurrent Endometrial Cancer	NCT03120624	I	Recruiting**
VSV-IFNβ-NIS	Cancer treatment	VSV-hIFNbeta-NIS in Treating Relapsed or Refractory Multiple Myeloma, Acute Myeloid Leukemia, or T-cell Lymphoma	NCT03017820	I	Recruiting**
rVSVΔG-ZEBOV-GP	Vaccine	Placebo-Controlled, Dose Response, Safety and Immunogenicity Study of Vesicular Stomatitis Virus (VSV) Ebola Vaccine in Healthy Adults (V920-004)	NCT02314923	I	Completed (75)
VSVΔG-ZEBOV	Vaccine	Safety and Immunogenicity of Prime-Boost Vesicular Stomatitis Virus (VSV) Ebola Vaccine in Healthy Adults (V920-002)	NCT02280408	I	Completed (76)
VSV-EBOV	Vaccine	Immune Durability After VSV-EBOV Vaccination	NCT02933931	I	Completed (77)
VSV-ZEBOV	Vaccine	VSV-ZEBOV Geneva Vaccine Trial	NCT02287480	II	Completed (78, 79)
VSV-Indiana (one type of VSV vector) HIV gag vaccine	Vaccine	Evaluating the Safety of and Immune Response to the VSV-Indiana HIV Vaccine in Healthy, HIV-Uninfected Adults	NCT01438606	I	Completed (80)
rVSVΔ-ZEBOV-GP	Vaccine	Phase I Trial of an Ebola Virus Vaccine (rVSVΔG-ZEBOV-GP)	NCT02283099	I	Completed (52, 78, 81)
rVSVΔG-ZEBOV	Vaccine	STRIVE (Sierra Leone Trial to Introduce a Vaccine Against Ebola)	NCT02378753	III	Completed (82–84)
VSVΔG-ZEBOV	Vaccine	Vaccine Treatment for Ebola Virus in Healthy Adults (V920-001)	NCT02269423	I	Completed (76)
rVSV-HIV1gag	Vaccine	Therapeutic Vaccine for HIV	NCT01859325	I	Completed (85)
rVSVN4CT1-EBOVGP1	Vaccine	Ebola Zaire Vaccine	NCT02718469	I	Completed (86)

**no publications, (published).

A decade ago, the first VSV trial in human cancers was posted to clinicaltrials.gov; however, no trial results have been disseminated (**Table 1**). This lack of information raises pertinent questions about whether we can achieve the desired therapeutic outcomes with current VSV vectors. Although it is not clear when these results will be available, the eagerly awaited outcomes of these studies will undoubtedly guide the future development of VSV-based oncotherapy for clinical translation. Nonetheless, several groups developing and testing oncolytic vesiculoviruses have proposed ingenious viral engineering strategies (65, 89, 90) to improve patient safety and vector potency.

## Strategies Designed to Enhance VSV Oncolytic Ability

Each genetic modification approach attempted to improve the antitumor activity of rVSV and its safety profile. Although rapid progress in nanotechnologies has enabled the improvement of delivery, pharmacokinetics, bioavailability in the tumor of rVSV vectors, many of these studies are in the early preclinical stages (91). Thus, this section will focus on vector engineering strategies to enhance safety, immunogenic apoptosis, and immune clearance.

### Optimizing rVSV Design to Enhance Immunogenic Apoptosis and Reduce Neurotoxicity

Studies have shown that mechanistically, VSV-induced oncolysis results in the release of a series of molecules, including tumor-associated antigens (TAAs), pathogen-associated molecular patterns (PAMPs), and damage-associated molecular patterns (DAMPs) (92–94). The build-up of TAAS in the tumor microenvironment elicits the recruitment and activation of tumor-specific cytotoxic (CD8+) T cells (92–94). PAMPS and DAMPS promote infiltration of neutrophils, natural killer (NK) cells, and dendritic cells (DC) into tumor sites. This simultaneous activation of innate and adaptive immunity is essential for priming a robust and durable antitumor immune response. These earlier works on the ability of rVSV to induce immunogenic cell death or apoptosis have influenced the field in many ways. For example, Wu and colleagues demonstrated that rVSV expressing murine gammaherpesvirus M3 protein (rVSV[M-Δ51]-M3) induced enhanced tumor necrosis and prolonged survival substantially in an animal model compared to parental VSV (19). M3 is a secreted chemokine-binding protein that binds to a broad range of mammalian chemokines with high affinity (19). In addition to decreasing neurotoxicity, delivery of exogenous M3 enhanced rVSV(M-Δ51)-M3 oncolytic activity by curtailing the activation of host innate immunity against oncolytic VSV in the tumor. Similar to the mutant M protein vectors, VSVs harboring mutations in G (95), P, or L proteins (96) with improved oncoselectivity and potency were also developed and evaluated preclinically. Although rVSVs with mutated viral proteins have enabled some safety improvements, as evidenced by no apparent neurovirulence and no visible pathogenesis in animal models, these vectors are often highly attenuated (i.e., reduce viral replication capacity) and thus are not appropriate for clinical deployment. Therefore, Russell and colleagues have adopted a different approach by incorporating microRNA target sequences (e.g., for miR-125) into the viral genome to decrease the ability of the virus to replicate in neurons (97).

### Generation of rVSV Vectors With Improved Immunostimulatory Activity

Multiple studies have also attempted to increase VSV safety and oncolytic properties by inserting into the viral genome genes encoding immunostimulatory proteins, chemoattractant molecules, or effectors that induce apoptosis in tumor cells (26, 59). One example is a VSV vector carrying the full-length p53 gene. Tumor protein p53 is a potent activator of apoptosis, and it is the most frequently mutated gene in human tumors (98). Indeed, the reactivation of p53 has been shown to potentiate antitumor immune activity (99). In an animal model, the vector VSV-M(Δ51)-p53, expressing p53, improved antitumor activity and enhanced CD49b+ NK and tumor-specific CD8+ T-cell responses (99, 100).

Immunostimulatory cytokines function in a synergistic or cascade fashion to modulate immune responses. Consequently, combining cytokines (101) with oncolytic viruses was seen as worth investigating for possible additive or synergistic long-term responses in clinical settings. Granulocyte–macrophage colony-stimulating factor (GM-CSF) is a potent immunostimulatory cytokine involved in the maturation and migration of macrophages and dendritic cells, which activate cytotoxic T cells (102). Hence, GM-CSF–expressing VSV (VSV-GM-CSF) vectors were developed, in which the transgene was inserted upstream of the VSV N gene or between the M and G genes (103–105). These vectors were attenuated and well-tolerated *in vivo*, and they triggered strong cellular and humoral antitumor immune responses (103–105). This work with VSV vectors expressing immunomodulatory cytokines demonstrated that tumor stage and type; immune mechanisms; and timing, dosage, and route of administration are crucial for obtaining the desired therapeutic effect with oncolytic viruses (106).

While IL-15 preferentially stimulates the proliferation of NK and memory CD8+ T cells and increases their antitumor activity (107), IL-12 functions as a “bridging” cytokine, providing an essential regulatory link between innate and adaptive immunity (108). Additionally, IL-23 has been shown to establish stable gene expression for activation of T_H_17 cells, but it is also crucial to activate innate immune cells, which are scattered across non-lymphoid organs (109). Thus, rVSV expressing IL-15, IL-12, or IL-23 (rVSV-IL-15, rVSV-IL-12, rVSV-IL-23) were generated and considerably improved synergistic antitumor efficacy compared to parental rVSV (110–112). Along the same line, IL-4 (113), thymidine kinase (113), IL-28 (114), Fms-like tyrosine kinase 3 ligand (115), and IFN-β (116) were expressed in VSV vectors, and their oncolytic activities have been documented across various cancer types. Based on encouraging preclinical studies, rVSV expressing human type-I IFN-β and a reporter known as sodium iodide symporter (VSV-IFNβ-NIS) has advanced through early to late phases of clinical testing (**Table 1**). Although VSV-based cytokine expression promotes superior oncolytic activity, it is essential to note that it can also potentiate viral clearance and impact the overall antitumor efficacy of the vectors.

### Addressing Issues With Rapid Immune Clearance, Dense Stroma While Promoting Strong Apoptotic Activity

A plethora of viral engineering strategies has been proposed to enhance the oncolytic ability of VSV. Chimeric VSV displaying fusion (F) and hemagglutinin (H) proteins from Newcastle disease virus or measles virus was shown to abolish VSV-associated neurotoxicity and the effect of virus-neutralization antibody (NAbs) on the bioavailability of viral vectors through the formation of syncytia-like structures (7, 57). The molecular mechanisms by which wild-type VSV and recombinant rVSV vectors induce intrinsic, extrinsic, or endoplasmic reticulum stress-mediated apoptosis have been elucidated in numerous studies (64, 65). This has prompted several authors to employ vectors such as VSV-vCKBPs (117), VSV-UL141 (118),, and rVSV-FAST (119), capable of exerting robust oncolytic activity while resisting rapid viral clearance. It is reasonable to speculate that many viruses in tumor sites could infect tumor cells and induce enough oncolysis to eradicate the tumors on their own. However, virus-mediated oncolysis also provides conditions for priming antitumor immunity by activating tumor-specific cytotoxic T cells (120–131). The most critical aspect of rVSVs in cancer vaccines’ context is their ability to efficiently modulate anti-tumor immune responses. Consequently, current oncolytic viruses, such as rVSV and rVSV-derived vectors, may be applicable to cancer patients with functional immune systems. In addition, the tumor microenvironment is complex, characterized by a sophisticated interplay between tumor cells and many components, including immune cells, extracellular matrix, fibroblasts, and various molecules, such as enzymes. This harsh environment is a known barrier to therapy, including immunotherapy and oncolytic virus therapy.^15^ It is now evident that rVSV vectors alone have limited long-term antitumor activity and may achieve only a partially curative effect. Combining rVSVs with other therapies, including radiotherapy, T-cell therapies, and immune checkpoint blockades, could serve to unleash their full oncolytic potential (132–138). We enthusiastically await the results of VSV clinical trials and expect novel combinations of VSV vectors with other cancer treatments to emerge in the coming years.

## Challenges and Future Directions

Despite evidence of the therapeutic benefits of rVSV-based oncotherapy, most investigations remain in the preclinical stage due to numerous challenges. These limitations include neurotoxicity (e.g., due to the promiscuous nature of the VSV entry glycoprotein [VSV-G]), rapid clearance by the immune system (e.g., *via* pre-existing VSV antibodies), and hepatotoxicity (e.g., viral interaction with Kupffer cells) (139, 140). Several strategies were proposed to address these obstacles, including modifying the VSV-G protein to achieve optimal therapeutic benefits (7, 53, 54, 141). Moreover, the lack of biomarkers that could be used to select patients who would benefit from oncolytic virus therapy represents a significant hurdle that we must seriously consider in future designs and clinical testing. Despite these challenges, the therapeutic potential of rVSV in cancer treatments is indisputable; indeed, VSV-IFN-β has advanced into late-phase clinical testing, renewing enthusiasm for oncolytic VSV.

## Discussion

Early research into the biology of VSV, including genomic structure, immunogenic properties, and pan-tropism, paved the way for developing this promising oncolytic agent and vaccine vector. However, the seamless clinical translation of VSV oncotherapy still faces significant challenges, and VSV has not yet been utilized to its full potential as an oncolytic vector. As the mechanisms of tumor resistance to molecular therapy continue to be elucidated, we fully expect new VSV vectors with enhanced potency and selectivity to be evaluated soon.

## Author Contributions

YZ and BN contributed to the study concept, drafting, and critical revision of the manuscript. Editorial support was provided by the Science Communication Group at the University of Arkansas for Medical Sciences. All authors approved the final, submitted version of the manuscript.

## Funding

This work was supported by the National Institutes of Health (NIH) through a National Cancer Institute (NCI) grant (CA234324 to BN) and start-up funds from the Winthrop P. Rockefeller Cancer Institute at the University of Arkansas for Medical Sciences to BN. The article contents are solely the responsibility of the authors and do not necessarily represent the official views of the NIH.

## Conflict of Interest

The authors declare that the research was conducted in the absence of any commercial or financial relationships that could be construed as a potential conflict of interest.

## Publisher’s Note

All claims expressed in this article are solely those of the authors and do not necessarily represent those of their affiliated organizations, or those of the publisher, the editors and the reviewers. Any product that may be evaluated in this article, or claim that may be made by its manufacturer, is not guaranteed or endorsed by the publisher.
